# Fabrication of the magnetic mesoporous silica Fe-MCM-41-A as efficient adsorbent: performance, kinetics and mechanism

**DOI:** 10.1038/s41598-021-81928-8

**Published:** 2021-01-28

**Authors:** Yige Guo, Bin Chen, Ying Zhao, Tianxue Yang

**Affiliations:** 1 College of Geology and Environment, Xi’an University of Science and Technology, Xi’an, 710054 China; 2 Shaanxi Provincial Center for Disease Control and Prevention, Xi’an, 710054 China; 3 State Key Laboratory of Environmental Criteria and Risk Assessment, Chinese Research Academy of Environmental Sciences, Beijing, 100012 China

**Keywords:** Nanoscale materials, Pollution remediation

## Abstract

Antibiotics are emerging pollutants and increasingly present in aquaculture and industrial wastewater. Due to their impact on the environment and health, their removal has recently become a significant concern. In this investigation, we synthesized nano zero-valent iron-loaded magnetic mesoporous silica (Fe-MCM-41-A) via precipitation and applied the adsorption of oxytetracycline (OTC) from an aqueous solution. The effects of competing ions such as Na^+^, Ca^2+^ and Cu^2+^ on the adsorption process under different pH conditions were studied in depth to providing a theoretical basis for the application of nanomaterials. The characterization of the obtained material through transmission electron microscopy demonstrates that the adsorbent possesses hexagonal channels, which facilitate mass transfer during adsorption. The loaded zero-valent iron made the magnetic, and was thus separated under an applied magnetic field. The adsorption of OTC onto Fe-MCM-41-A is rapid and obeys the pseudo-second-order kinetic model, and the maximum adsorption capacity of OTC is 625.90 mg g^−1^. The reaction between OTC and Fe-MCM-41-A was inner complexation and was less affected by the Na^+^. The effect of Ca^2+^ on the adsorption was small under acidic and neutral conditions. However, the promotion effect of Ca^2+^ increased by the increase of pH. Cu^2+^ decreased the removal efficiencies continuously and the inhibitory effects decrease varied with the increase of pH. We propose that surface complexing, ion-exchange, cationic π-bonding, hydrogen bonding, and hydrophobicity are responsible for the adsorption of OTC onto Fe-MCM-41-A.

## Introduction

Water is our most necessary resource. It is also becoming a scarce resource in many parts of the world. The pollutants in our water are also becoming more difficult to remove. Levels and kinds of antibiotics in our drinking water are all on the rise^1^. Oxytetracycline (OTC), as a broad-spectrum antibiotic, is extensively used in human therapy, livestock breeding and aquaculture^2,3^. Recent studies indicate that the OTC is roughly 100–340 ng L^−1^ in surface water^4,5^, for example, the maximum detected and mean concentrations of oxytetracycline in Honghu lake, China are up to 2796.6 and 161.9 ng L^−1^ ^6^. The continuous presence of OTC in environment can harm the growth of plants and animals and cause resistance genes^7^.

Oxytetracycline (OTC) is difficult to degrade owing to its toxicity and resistance to microorganism^8^. The elimination of OTC in few of the biological treatments has been distinguished by quick sorption and slow biodegradation^9^. Traditional water treatment technologies have a limited removal efficiency of 20–30% for antibiotics^10^. Besides, antibiotic pollution is characterized by pH dependence and heavy metal contamination^11^. OTC is a complex organic compound and show positive, zwitterion or negative charges vary with pH. The active groups of OTC will combine with metal ions to form complex pollutants, which will affect each other's characteristics and enhance toxic effects^12^. Therefore, the demand for effective removal of OTC is urgent.

Treatment techniques based on physical and chemical methods, such as adsorption, are the forefront of water remediation^13^. In the past few decades, the emergence of nanomaterials has greatly promoted the development of water treatment technology^14,15^. MCM-41 is a silica-based material which can be adopted as an adsorbent owing to its larger surface area, flexible pore structure, larger pore volume, and nontoxicity^16^. The hexagonal phase channel expedites the adsorption of oxytetracycline, a polycyclic macromolecule. The nanoscale pore channels inside the material can provide a large number of adsorption reaction micro-interfaces, which can change the material and energy transfer process on the interface, promote concentration accumulation of pollutants, reaction activation, and advance the reaction^17^.

The MCM-41 has been assembled with zeolites (e.g., zeolite A, zeolite X), iron oxide nanoparticles and et al. to enhance its performance^18–20^. Nanoscale zero-valent iron (NZVI) has recently drawn rising attention for water pollution remediation due to its high activity and separability^21^. However, to the best of the author’s knowledge, little information has been reported on the performance and mechanism of removal of OTC from water using NZVI-loaded MCM-41-zeolite composites, which is the objective of this investigation.

Herein, an efficient method was developed for the remediation of OTC. The specific objectives were (1) to synthesize and characterize nano zero-valent iron (NZVI)-loaded magnetic mesoporous silica (Fe-MCM-41-A); (2) to examine OTC removal performance under different conditions, i.e., initial solution pH initial concentrations, adsorbent dosages and competition ions; (3) to carry out the adsorption kinetics and thermodynamics analysis; and (4) to elucidate the possible adsorption mechanisms for the removal of OTC.

## Materials and methods

### Materials

OTC (> 97% purity), Na_2_O_3_Si·9H_2_O (AR), NaAlO_2_ (AR) were obtained from Shanghai Minrell Chemical Technology Co., LTD. NaOH (AR), NaBH_4_ (> 98% purity), C_19_H_42_BrN ((CTAB) AR), FeCl_3_ 6H_2_O (AR), NaCl (AR), CaCl_2_ (AR), CuCl_2_ (AR), HCl (99 wt.%), C_2_H_2_O_4_ (AR), C_2_H_5_OH (AR), C_2_H_3_N (GR) and HNO_3_ (AR) were supplied by Sinopharm Chemical Reagent Co., Ltd (China). Deionized water was used in all experiments.

### Preparation of Fe-MCM-41-A

We synthesized MCM-41-A through impregnation-assisted one-step crystallization method^22^. The specific preparation process was described in the Supplementary information. Fe-MCM-41-A and NZVI were synthesized according to the previously reported precipitation method^8^. MCM-41-A (4.0 g) and FeCl_3_.6H_2_O (19.32 g) were dispersed in 100 mL degassed C_2_H_5_OH solution (80 wt%) after 30 min of magnetically stirring 200 mL of 1 M NaBH_4_ (approximately 4–5 mL min^−1^) was added dropwise under stirring. All procedures were conducted under N_2_ environment. The obtained solid suspension was rinsed more than three times with ethanol, then drying to constant weight under vacuum condition at 50 °C. NZVI was synthesized by the above steps without MCM-41-A.

### Adsorbents characterization

The morphology of Fe-MCM-41-A was observed by TEM (Tecnai G2 F20). The BET measurements and pore-size distribution were performed (Specific Surface Area Analyzers), the crystal structure of Fe-MCM-41-A was characterized by XRD (Ulitma IV), UV–Vis DRS, FT-IR (TENSOR 37), XPS (Axis Ultra DLD), Mossbauer analysis and magnetization analyses were further employed to characterize the obtained samples.

### Batch experiments

#### Adsorption efficiency experiments

The adsorption efficiency experiment was carried out by single factor experiment^8^. Adsorption studies were conducted at different contact times, pH, and initial concentrations to assess the potential removal of OTC. Moreover, the adsorption mechanisms have been presented based on the achieved results. The specific experimental steps were described in the Supplementary information.

#### Competitive ion experiments

A gradient series of concentrations of Na^+^ or Ca^2+^ solutions (0.01, 0.02, 0.05, 0.1 M) and Cu^2+^ solutions (5.0, 10.0, 20.0 and 50.0 mg L^−1^) were prepared and used as background solutions to obtain OTC solution. The other steps are the same as the adsorption efficiency experiments as showed in Supplementary information. Computational details were described in the Supplementary information.

#### Regeneration experiments

The exhausted Fe-MCM-41-A was regenerated by Ultrasonic reduction method. The OTC was desorbed through ultrasonic method and the reactivity of Fe-MCM-41-A was recovered by reduction of sodium borohydride. The specific experimental steps were described in the Supplementary information.

## Results and discussion

### Characterization of Fe-MCM-41-A

**Figure 1:**
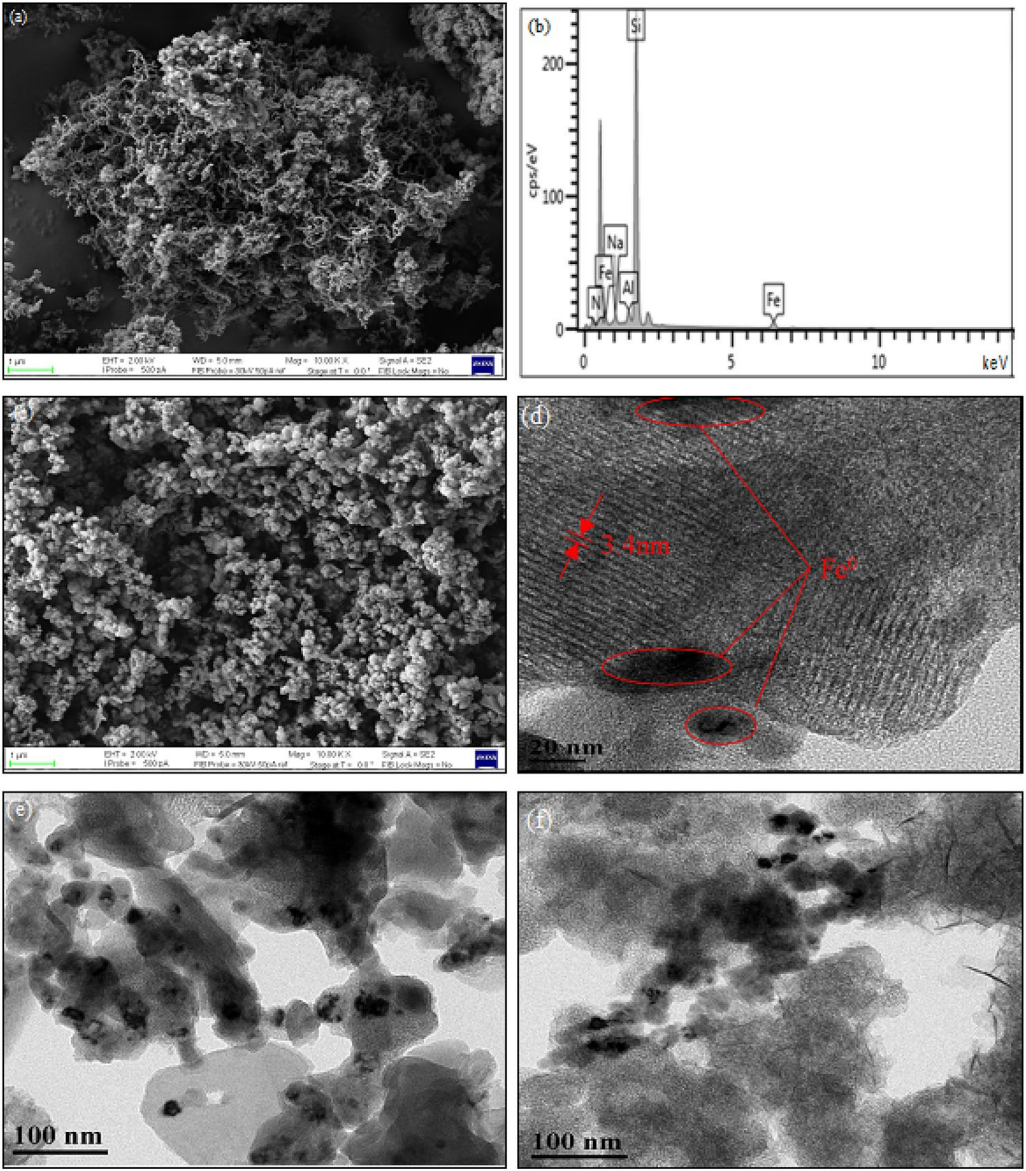
(**a**) SEM and (**b**) EDX of Fe-MCM-41-A, (**c**) SEM of OTC-Fe-MCM-41-A, (**d**) HRTEM of Fe-MCM-41-A, (**e**) TEM of Fe-MCM-41-A and (**f**) OTC-Fe-MCM-41-A.

The SEM image and EDX of Fe-MCM-41-A showed in Fig. 1a,b demonstrates the linear nano-structure of the material and that the sample has loose texture and uniform structure. According to the HRTEM and TEM images (Fig. 1d,e), it can be seen that the sample exhibited a hexagonal pore structure and Fe^0^ is uniformly loaded in the sample. These results demonstrate that the mesopore structure was not undermined by Fe loading. Figure 1d shows that the sample has an ordered arrangement of hexagonal channels with uniform pore size of 3.4 nm. This indicates that the material keeps the pore structure well after the loaded of zero-valent iron. This mesoporous structure of the material is conducive to the mass transfer process of the adsorption reaction and the increase of the adsorption capacity.

**Figure 2:**
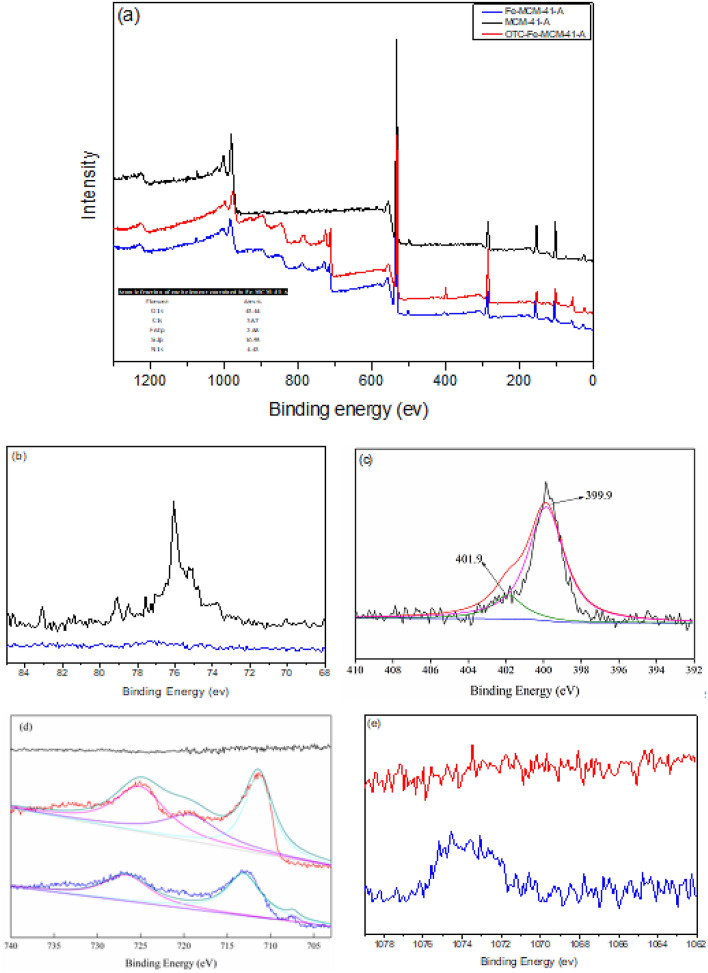
(**a**) Full survey XPS spectra of MCM-41-A, Fe-MCM-41-A and OTC-Fe-MCM-41-A. The XPS spectra of (**b**) Al in MCM-41-A and Fe-MCM-41-A, (**c**) N1s in OTC-Fe-MCM-41-A, (**d**) Fe2p in MCM-41-A, Fe-MCM-41-A and OTC-Fe-MCM-41-A, (**e**) Na in Fe-MCM-41-A and OTC-Fe-MCM-41-A.

As indicated in Fig. 1S, the surface area and average pore size of Fe-MCM-41-A is 360.4 m^2^ g^−1^ and 3.4 nm, respectively, which is consistent with the analysis of TEM. The small angle (0.7°–10°) XRD analysis of Fe-MCM-41-A (Fig. 2S) show the characteristic diffraction peaks at 2θ of 2.1° and 4.2° represent the (110) and (200), planes of Fe-MCM-41-A with hexagonal mesoporous crystal structure, respectively^23^. The 2θ peak at 44.9° designates that Fe in the material is mainly zero-valent^24^. The small peak at 37° represents iron oxidation which may be due to the oxidation of Fe0 during preservation.

It can be seen from Fig. 2b that the characteristic peak of Al in Fe-MCM-41-A decreases significantly, while the characteristic peak of Fe in Fe-MCM-41-A increases accordingly. This demonstrated that the content of Al in MCM-41-A decreases and the content of Fe element increases after the loading modification of NZVI. It can be seen from Fig. 2d that photoelectron peaks appear in the Fe 2p3/2 spectra. The peak of 707.1 eV corresponds to the fe2p3/2 electron orbital peak of Fe^0^^25^, indicated that the sample in freshly synthesized contains zero valent iron. This analysis is consistent with the XRD analysis. So, we speculated that a decrease in the Al content of Fe-MCM-41-A during modification may be due to the replacement of Fe in the skeleton of MCM-41-A^26^.

**Figure 3:**
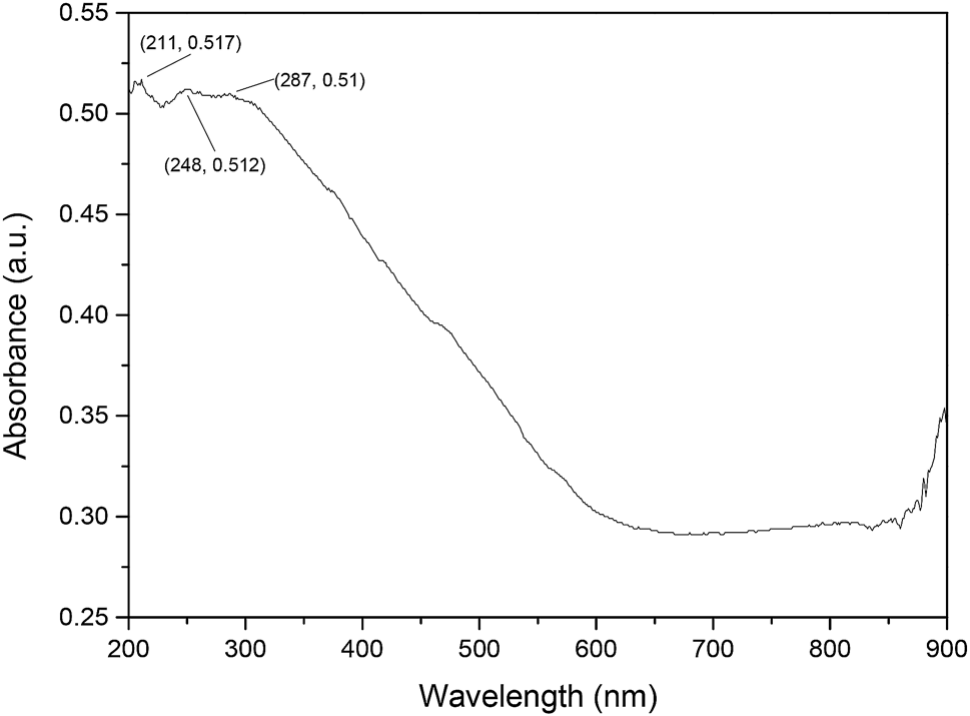
UV–Vis DRS spectrum of Fe-MCM-41-A.

**Figure 4:**
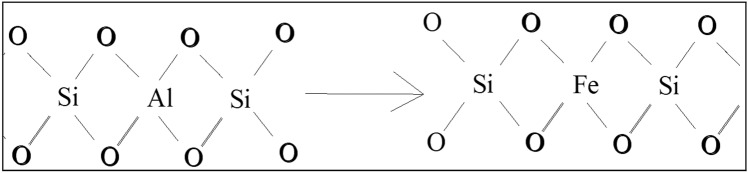
The replacement of Fe in cage construction of Fe-MCM-41-A.

To further investigate the nature and binding form of Fe in Fe-MCM-41-A, DRS UV–Vis spectroscopy experiments were conducted and the results are shown in Fig. 3. Silicon based multihole materials have no bands in 200–270 nm region^27^ and the absorption bands at 287 nm may be contributed by the pore structure of Fe-MCM-41-A^28^. However, the sample shows absorption bands centered at 210–250 nm, this indicates that Fe has been dispersed within the material. The bands at 211 nm and 248 nm may be assigned to the d¼–p¼ charge-transfer transition between the iron and oxygen atoms in the framework of Fe–O–Si in Fe-MCM-41-A^29^. So, we assume that the schematic diagram of displacement reaction in the modification process as shown in Fig. 4.

**Figure 5:**
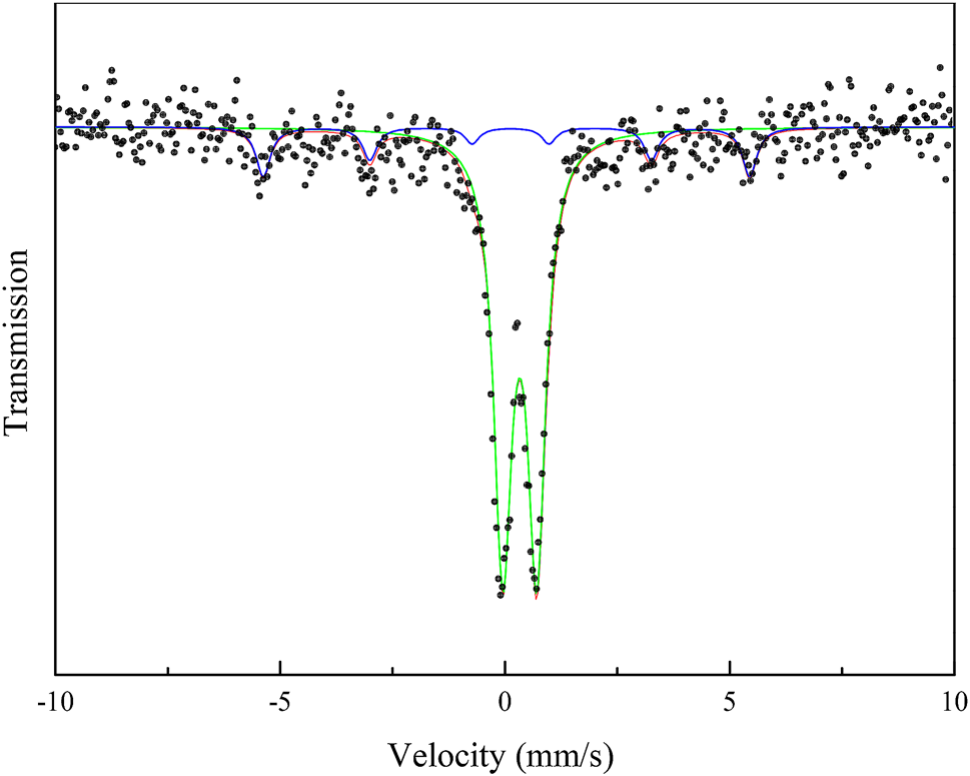
Mossbauer spectrum of Fe-MCM-41-A at room temperature.

Figure 5 shows the Mössbauer spectrum of Fe-MCM-41-A, and this spectrum is a superposition of one major paramagnetic doublet and one minor broad magnetic sextet. The six-line pattern of the spectrum is characteristic of the body-centered cubic face of metallic α-Fe confirmed by the hyperfine interaction parameter values shown in Table 1. The major paramagnetic doublet indicating that the paramagnetic state of the sample^30^, therefore, the agglomeration of Fe-MCM-41-A caused by excessive residual magnetism can be avoided. The small contribution of the sextet may be due to refinements of the structure to the nanometer level of Fe. As shown in Table 1, the doublet (IS of 0.33 mm/s and QS of 0.76 mm/s) indicates the positively charged Fe in the sample^31^, the sextet (IS of 0.081 mm/s and QS of − 0.046 mm/s) indicates the existence of Fe0^32^. Since the sample was subjected to Mössbauer analysis more than 2 months after its preparation, the sample may be partially oxidized which is consistent with the XPS analysis.

**Table 1:** Hyperfine interaction parameters of Fe-MCM-41-A measured by Mössbauer spectroscopy.

Component/compound	IS(mm s^−1^)	QS (mm s^−1^)	B_hf_ (KOe)	Γ (mm s^−1^)
Sextet/iron	0.330 (11)	0.755 (19)	–	0.244 (14)
Doublet/sextetiron	0.081 (66)	− 0.046 (66)	335.5(45)	0.193 (98)

Uncertainty of the values is given in parentheses for the last significant number. IS isomer shift relative to α-iron, QS quadrupole splitting for the doublet and quadrupole shift for the sextet, B_hf_ hyperfine magnetic field, Γ half width at half maximum of spectral lines.

**Figure 6:**
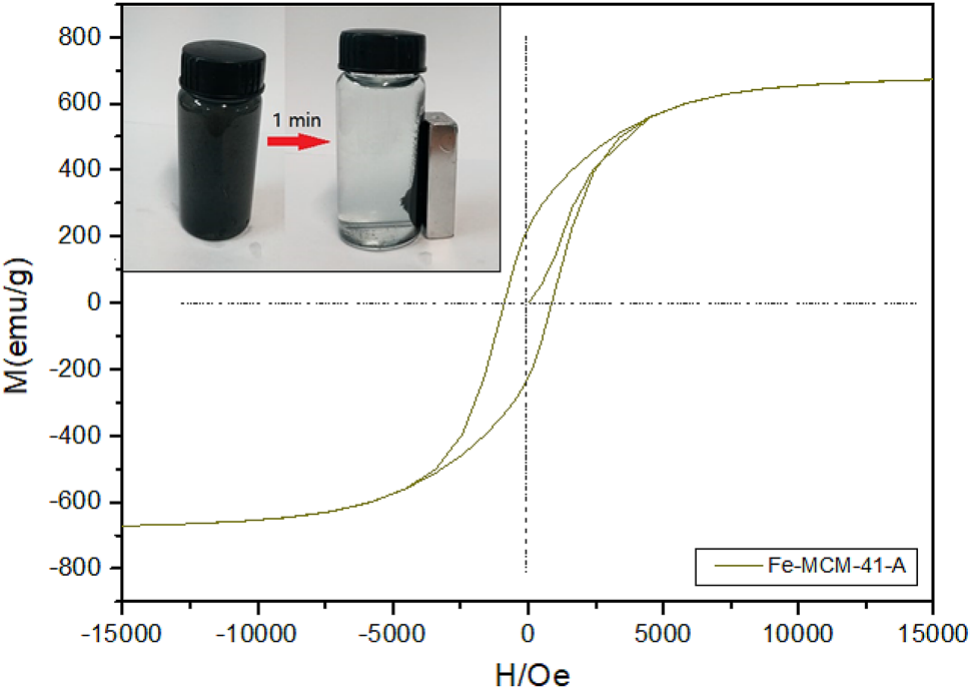
Hysteresis loop of Fe-MCM-41-A and separation effect applied magnetic field in 1 min.

The zero-valent iron loaded material is magnetic and can be separated quickly under applied magnetic field. The magnetization properties of Fe-MCM-41-A were analyzed by hysteresis loop test. Figure 6 shows that Fe-MCM-41-A is strongly magnetic and can be separated within 1 min under applied magnetic field.

### Adsorption performance

#### OTC removal efficiency using NZVI, MCM-41-A and Fe-MCM-41-A

**Figure 7:**
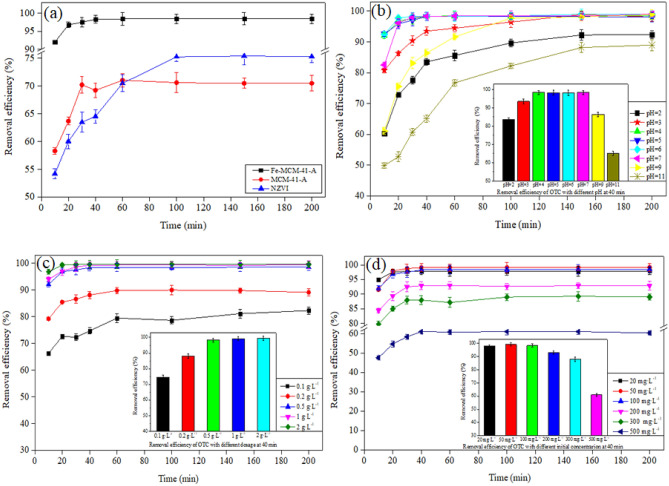
(**a**) Comparison of NZVI, MCM-41-A and Fe-MCM-41-A in OTC removal, C_0_: 100 mg L^−1^; dosage: 0.5 g L^−1^; T: 25 °C. Removal efficiency of OTC onto Fe-MCM-41-A in different conditions: (**b**) C_0_: 100 mg L^−1^; dosage: 0.5 g L^−1^; T: 25 °C, (**c**) C_0_: 100 mg L^−1^; pH 5.0; T: 25 °C, (**d**) dosage: 0.5 g L^−1^; pH 5.0; T: 25 °C.

Comparison of NZVI, MCM-41-A and Fe-MCM-41-A in OTC removal was shown in Fig. 7a. The adsorption of OTC onto Fe-MCM-41-A and McM-41-a reach an equilibrium in 60 min, the OTC removal rate was 98.4% and 71.04%, respectively. However, the adsorption of OTC onto NZVI reached equilibrium in ~ 100–150 min, with a removal rate of 75.2%. The rapid adsorption of OTC onto Fe-MCM-41-A and MCM-41-A is mainly due to the cellular mesoporous structure that facilitates mass transfer^17,33^, the large specific surface area and the charged surface of the material^34^. Moreover, the OTC removal rate of Fe-MCM-41-A is higher than that of MCM-41-A, indicating that modification of MCM-41-a by NZVI load is conducive to improving the adsorption performance.

#### Removal kinetics

The adsorption of OTC onto Fe-MCM-41-A is rapid especially in the first 20 min (Fig. 7a). This high adsorption was mainly due to the large surface area, hexagonal mesoporous structure and appropriate pore size of Fe-MCM-41-A which promote internal mass transfer during the adsorption^35^. In this work, the adsorption capacity of OTC was 196.8 mg g^−1^, when 100 mg L^−1^ OTC solution was remediated with Fe-MCM-41-A (0.5 g L^−1^) at pH 5.0. Correlation coefficient (*R*^2^ = 0.9999) indicates that the adsorption obeys the pseudo-second-order kinetic model (Table 2). This indicates that the removal of OTC was rate-controlling limiting step.

**Table 2:** Parameters in adsorption kinetic equation of OTC onto Fe-MCM-41-A.

Kinetic model	Equation	Parameters	Value
Pseudo-first-order	log(q_e_ − q_t_) = logq_e_ − k_1_/2.303t	k_1_ (min^−1^)	0.1011
		q_e_ (mg g^−1^)	30.49
		R^2^	0.9792
Pseudo-second-order	(t/q_t_) = 1/(k_2_q_e_) + 1/q_e_(t)	k_2_ (g mg^−1^ min^−1^)	1.6531
		q_e_ (mg g^−1^)	197.94
		R^2^	0.9998

#### Effect of pH

Figure 7b reveals that from pH 2.0 to 5.0, the removal efficiency increased from 92.2 to 98.4%, and then from pH 5.0 to 11.0, the efficiency decreased from 98.4 to 76.7%. This proves that a weak acidic condition could favor the adsorption process. The adsorption of OTC onto Fe-MCM-41-A reached equilibrium in 40 min at pH 4.0 and 5.0; however, the adsorption lasted for more than 150 min at pH 2.0 and 11.0. This could be due to the digestion of Fe and the desilication of Fe-MCM-41-A under strong acidic and alkaline conditions. The highest adsorption efficiency was achieved at pH 5.0. When the pH is higher than 7.4 (pK_a2_), HL^−^ and L^2−^ become predominant species and Kow of OTC also decreases, thereby Fe-MCM-41-A surfaces become negative simultaneously. By this, we could infer that a decrease in the removal efficiency at pH 11.0 might be largely due to the weakening of hydrophobic function^35^.

#### Effect of adsorbent dosage

Figure 7c exhibits that the removal efficiency of OTC increased with the amount of adsorbent. When the dosage reached 0.5 g L^−1^, the removal efficiency no longer increased with the amount of adsorbent owing to agglomeration and particle precipitation of Fe-MCM-41-A at high solid to liquid ratio.

#### Effect of initial OTC concentration

It has been noticed that the initial concentration of OTC can significantly influence the removal efficiency. Figure 7d shows that the adsorption capacity of OTC increased and the removal efficiency increased at first and then decreased with an increase in the initial concentration. The highest removal efficiency was 99.1% at an initial concentration of 50 mg L^−1^, and the maximum equilibrium adsorption capacity was 609.0 mg g^−1^ at an initial concentration of 500 mg L^−1^. The main reason for the decrease of adsorption efficiency was the lack of adsorption sites. The enhancement of the adsorption capacity was due to the high driving force caused by the high concentration gradient. Besides, at high initial OTC concentrations, a large number of OTC were distributed around Fe-MCM-41-A, complex [Fe(TC)]_n_^3+^ were formed and deposited on the surface of the adsorbent instantaneously, thus might block the mass transfer channels^36^. The higher initial OTC concentration led to faster formation of [Fe(TC)]_n_^3+^, hence, the longer reaction equilibrium time.

#### Effect of ions

**Figure 8:**
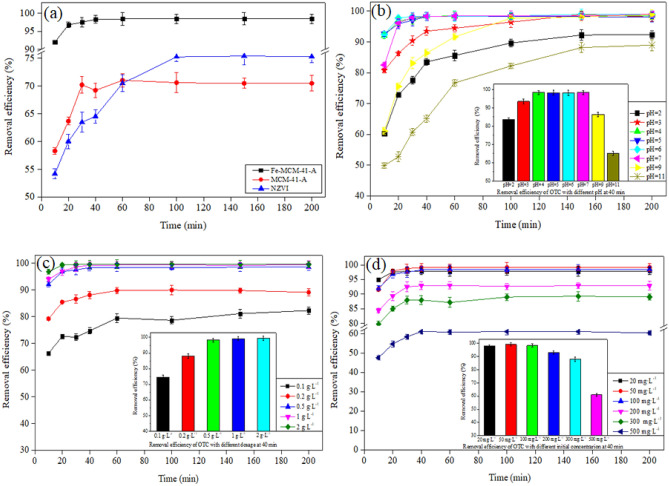
Effects of ionic strength on the removal efficiency of OTC onto Fe-MCM-41-A under varied pH (**a**) Na^+^, (**b**) Ca^2+^, (**c**) Cu^2+^ and (**d**) removal efficiency of Cu^2+^.

Figure 8 shows that the removal efficiency of OTC reduced less than 7% while the concentration of Na^+^ in background solution increased from 0.0 M to 0.1 M in each pH condition. The adsorption was less affected by the Na^+^, this was owing to the inner complexation between OTC and Fe-MCM-41-A^37^, as inner layer complexation can be hardly affected by the ionic strength in the background solution^38,39^.

Figure 8 shows that the effect of Ca^2+^ on the adsorption was small under acidic and neutral conditions. However, the removal efficiency increased with the increasing of the concentration of Ca^2+^ under alkaline condition, and the promotion effect increased by the increase of pH. This was mainly benefit from the bridging effect of Ca^2+^, OTC combined with Ca^2+^ to form TCs-Ca^2+^-Fe-MCM-41-A^40^.

Another reason for the removal efficiency of TCs increased with the increasing of Ca^2+^ concentration under alkaline condition was the effect of surface chemical precipitation. TCs molecules may complex with Ca^2+^ and deposited on the surface of Fe-MCM-41-A^41^. Besides Ca^2+^ could reduce the hydrophilicity of OTC and helped to prompt TCs adsorbed onto Fe-MCM-41-A under hydrophobic forces^42^.

The removal efficiencies of TCs onto Fe-MCM-41-A decreased continuously with the increase of Cu^2+^ (Fig. 8), meanwhile, the inhibitory effects of Cu^2+^ decrease varied with the increase of pH. The inhibiting effect of Cu^2+^ was mainly due to the complexation between Cu^2+^ and TCs. The surface charge of OTC increased after complexation with Cu^2+^ and the electrostatic repulsion between OTC and Fe-MCM-41-A also increased, which may inhibit the removal of OTC.

#### Adsorption isotherm

According to the adsorption isotherms analysis on the removal of OTC, we found that the equilibrium concentration increased along with the amount of OTC adsorbed onto Fe-MCM-41-A. The adsorption of OTC onto Fe-MCM-41-A obeys the Langmuir model (Table 3). The constant *q*_max_ at 25 °C obtained from Langmuir model was 625.90 mg g^−1^. This indicates that the adsorption of OTC onto Fe-MCM-41-A follows monolayer adsorption which occurs on a relatively uniform surface.

**Table 3:** Isotherm constant parameters and R^2^ calculated for the adsorption of OTC onto Fe-MCM-41-A.

Adsorption isotherm model	Equation	Parameters	Value
Langmuir	$\frac{{C_{e} }}{{q_{e} }} = \frac{1}{{q_{\max } k}} + \frac{1}{{q_{\max } }}C_{e}$	k (L mg^−1^)	0.1758
		Q_max_ (mg g^−1^)	625.90
		R_L_	0.0538
		R^2^	0.9989
Freundlich	$\log q_{e} = \log K_{{\text{f}}} + \frac{1}{n}\log C_{e}$	K_f_ (mg g^−1^)	107.4484
		1/n	0.3929
		R^2^	0.8485

### Adsorption mechanism

**Figure 9:**
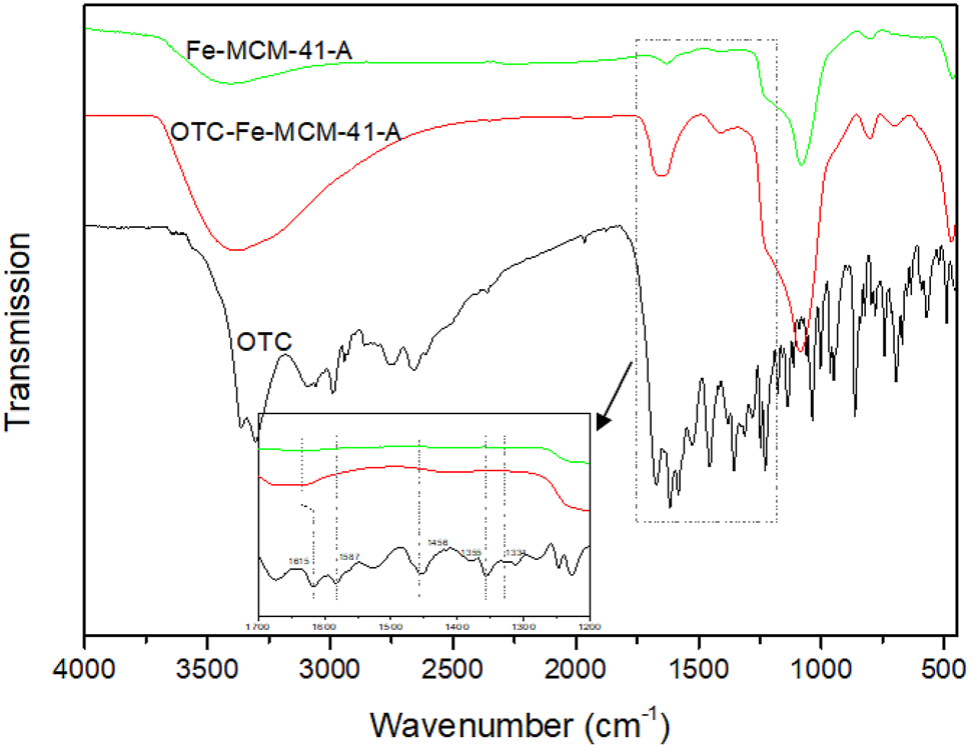
FT-IR spectrograms of Fe-MCM-41-A, OTC-Fe-MCM-41-A and OTC.

The functional groups of Fe-MCM-41-A, OTC-Fe-MCM-41-A and OTC were measured by FT-IR. As shown in Fig. 9, the characteristic peaks of OTC are principally placed at 1200–1700 cm^−1^. Peaks at 1615 cm^−1^, 1587 cm^−1^,1458 cm^−1^ and 1366 cm^−1^ can be attributed to attributed to a stretching of the C=O groups, C–H bonds, amide-NH groups and C–N bonds, respectively^43,44^. The typical bands of C–N and amide-NH shifted to higher frequencies after adsorption, indicating the reaction between amide groups in OTC with Fe-MCM-41-A. The peak located in 3200–3500 cm^−1^ can be assigned to –OH band. The broadening and peak of –OH band strength weakening after the adsorption, indicated that the –OH band in OTC changed from free state to association state^45^. This may be due to the hydrogen bonding between neutral -OH band and non-ionized –Si–OH band in TCs^46^.

XPS were further employed to study the adsorption mechanism (Fig. 2), the results of analysis prove that the intensities of N 1s increased, whereas the intensities of Na 1s decreased after adsorption. This could be due to that parts of OTC adsorb onto Fe-MCM-41-A through ion-exchange. In Fig. 2c the peaks at 399.9 eV and 401.9 eV could be correspond to the amino group and its disturbation caused by Fe after adsorption, respectively, indicated that Fe-MCM-41-A could be combined with the amino bands in OTC by cationic—bonding. The Fe2p_3/2_ and Fe2p_1/2_ spectra emerged at the binding energies of 725.2 eV and 711.4 eV, respectively, the chemical shift in the spin–orbit coupling was 13.8 eV (Fig. 2d). This suggests that the cationic-bond between Fe-MCM-41-A and -NH on OTC could be formed through polarization^47^.

Besides, according to the law of removal efficiency and octanol–water distribution coefficient (Kow) of OTC, it can be inferred that an increase in the hydrophobicity of OTC is conducive to the adsorption of OTC on Fe-MCM-41-A.

### Comparison of various adsorbent

Performances of different materials for the adsorption of OTC were compared in Table 4. The q_max_ of OTC onto Fe-MCM-41-A was higher than other similar materials. This honeycomb-like magnetic mesoporous silica Fe-MCM-41-A synthesized in this study was superior to other materials in both adsorption rate and efficiency.

**Table 4:** Comparison of removal capacities of OTC with various materials.

Adsorbate	Adsorbent	Time (h)	Dosage (g L^−1^)	pH	*T* ( °C )	*q*_*max*_ (mg g^−1^)	References
OTC	Undecenoic acid-coated MNPs	–	–	5.0	15	86.96	^48^
	MIL-101(HCl)	1	–	–	25	44.7	^49^
		1	–	–	5	115.34	^49^
	Activated sludge	–	–	5	20	90.9	^50^
	Activated carbon Sorbo Norit	192	1	4–5	25	252.6	^51^
	Activated carbon Merck	192	1	4–5	25	413.2	^52^
	Cyclodextrin Polymer	0.5	20	4–7	25	1.2	^53^
	MWCNT	100	–	7	23	73	^54^
	Fe-MCM-41-A	1	0.5	5	25	625.9	This work

### Regeneration of Fe-MCM-41-A

**Figure 10:**
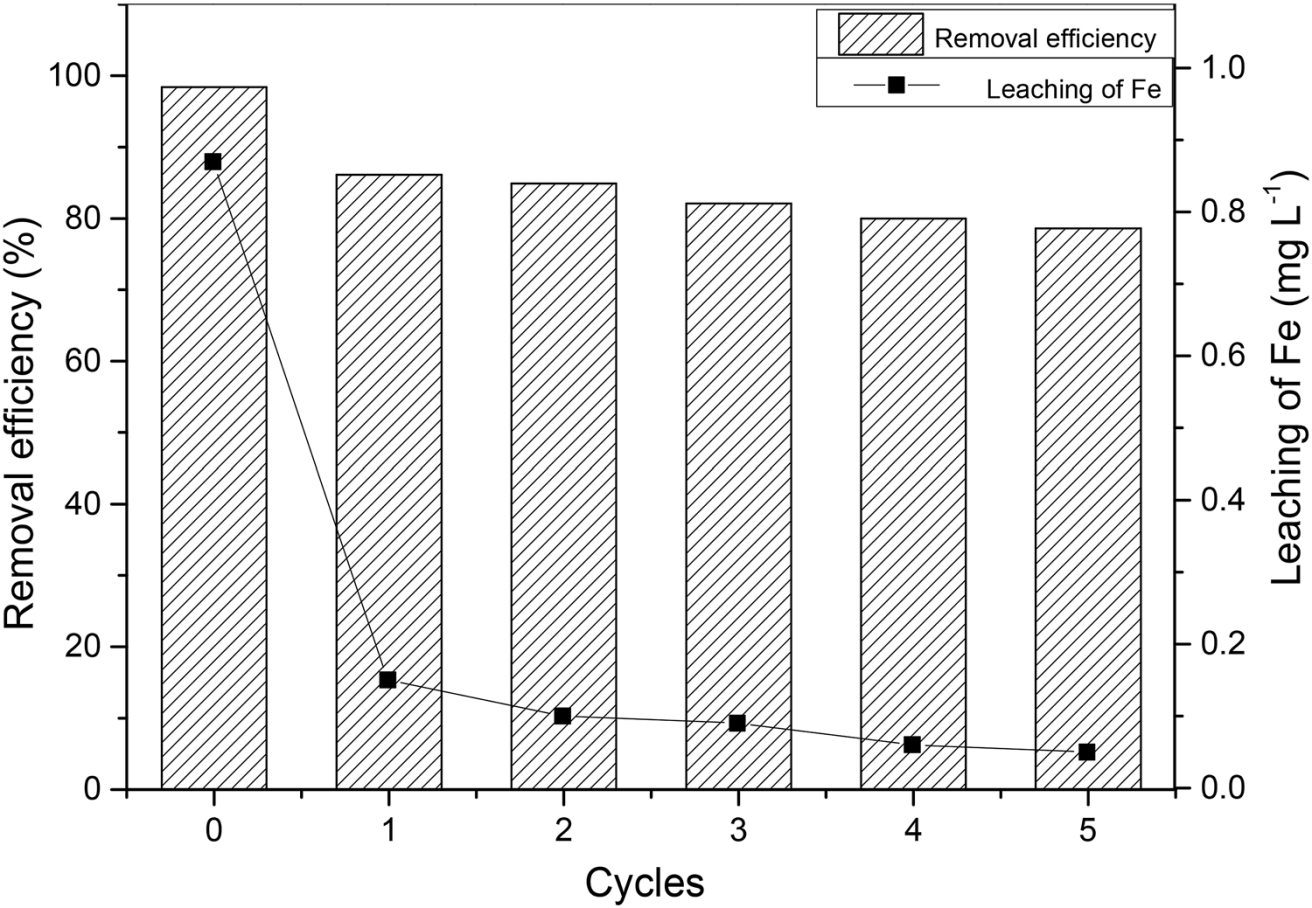
Removal efficiency of Fe-MCM-41-A after repeated regeneration and iron leaching after repeated regeneration for five cycles (pH 5.0, C_0_: 100 mg L^−1^; dosage: 0.5 g L^−1^; T: 25 °C).

In the regeneration experiment, the removal rate remained above 78% (Fig. 10) after five rounds of sorption–desorption cycles, indicating that Fe-MCM-41-A has a good regenerability. From Fig. 1c and f it can be seen that after the adsorption of OTC the adsorbent retains the original morphology and pore structure well, which also proves that the structural stable material is renewable. Leaching of iron was investigated after each cycle as an indication of the stability of Fe-MCM-41-A. As shown in Fig. 10, the amount of leached iron ions was the highest in the first cycle (0.87 mg L^−1^). After third cycle, leached iron reduced to < 0.09 mg L^−1^ and reasonably stable for the subsequent cycles reducing to 0.05 mg L^−1^ at the fifth cycle. The change law of iron loss is consistent with that of adsorbent regeneration property, so we assumed that this trivial iron loss is an important factor for reuse of the adsorbent. The decrease in removal efficiency occurred mainly in the second cycle, this might have been caused by an irreversible occupation of partial-adsorption sites and the loss of nano-absorbent, as has been observed in previous studies^8^.

## Conclusions

A kind of magnetic hexagonal mesoporous silica Fe-MCM-41-A was synthesized and demonstrated to be an efficient adsorbent for the removal of OTC from aqueous solution. The ordered hexagonal pore structure of Fe-MCM-41-A which can facilitate mass transfer was observed by TEM and XRD analysis. The adsorption of OTC onto Fe-MCM-41-A is rapid and obeys the pseudo-second-order kinetic model. The obtained value of *q*_max_ according to Langmuir model was 625.90 mg g^−1^. We propose that surface complexing, ion-exchange, cationic π-bonding, hydrogen bonding, and hydrophobicity are responsible for the adsorption of OTC onto Fe-MCM-41-A.

## Supplementary Information

Supplementary Information.

## Data Availability

The datasets generated during and/or analysed during the current study are available from the corresponding authors on reasonable request.
