# Supplementary material for: Fabrication of the magnetic mesoporous silica Fe-MCM-41-A as efficient adsorbent: performance, kinetics and mechanism
Source: Sci Rep. 2021 Jan 28;11:2612. doi: 10.1038/s41598-021-81928-8 (PMC7843643; doi:10.1038/s41598-021-81928-8)
Supplement: Supplementary file 1 — Supplementary Information. [file 41598_2021_81928_MOESM1_ESM.pdf]

Manuscript Title: Fabrication of the magnetic mesoporous silica Fe-MCM-41-A as efficient adsorbent: Performance, kinetics and mechanism

Authors information: Yige Guo[1], Bin Chen[2*], Ying Zhao[3], Tianxue Yang[3*]

(1 College of Geology and Environment, Xian University of Science and Technology, Xian, 710054, China. 2 Shaanxi provincial Center for Disease Control and Prevention, Xian 710054, China. 3 State Key Laboratory of Environmental Criteria and Risk Assessment, Chinese Research Academy of Environmental Sciences, Beijing, 100012, China. Correspondence to chenbin6121@163.com or 2316378041@qq.com)

Supplementary material:

1. Preparation of MCM-41-A

MCM-41-zeolite A (MCM-41-A) samples were synthesized using the impregnation assisted one-step crystallization method. Firstly, zeolite A precursors were prepared by mixing NaOH, $NaAlO_2$ and Na_2SiO_3•$9H_2O$ in a beaker. The molar ratio of SiO_2: Al_2O_3: H_2O: Na_2O was set at 1: 0.83: 150: 1.15, these chemicals were added into the boiling water, stirred vigorously for 1h, and then aged at room temperature. Secondly, solution A was prepared by dissolving 4.37 g of CTAB into 30 ml of water; Solution B was prepared by dissolving 23.6 g of Na_2SiO_3•$9H_2O$, 0.96 g of NaOH and 0.4 g of $NaAlO_2$ into 40 ml of water. Solutions A and B were then thoroughly mixed and added to 1% weight of zeolite A precursors. After 1h of vigorous stirring, the solution pH was adjusted to 10.5, and the mixture was transferred to a 200 mL polytetrafluoroethylene autoclave, crystallized at 105°C under constant stirring (30

rpm) for 48 h. The obtained solid product was filtered, rinsed with water until the washed water became neutral, then dried at 105°C for 12 h. Finally, the dried powder was calcined in air at 550°C for 4 h at a temperature ramping rate of 5°C min^{-1} to obtain MCM-41-A.

2. Batch Experiments

Batch experiments were performed using a series of 50 mL bottles covered with tin foil paper (avoid the photolysis of OTC). Fresh OTC stock solution (500 mg L^{-1}) was prepared every time through adding a certain amount of OTC into distilled water. The pH values were adjusted by HCL or NaOH solutions. Then the solutions were mixed with 0.05 g of prepared Fe-MCM-41-A (except when the effect of dosage was investigated). The bottles were plugged with stoppers and continuously shaken (200 rpm) at 25°C (except when the effect of temperature was investigated). Sample solutions were filtered through a PES (polyethersulfone) micro-filtrate membrane (0.45 um), and the OTC concentration was measured via high performance liquid chromatography (HPLC). Column: Pgrandsil-STC-C18, 5 μm, 100 Å, 4.6 mm × 250 mm; 0.01 M oxalic acid–acetonitrile (77:23, V/V) as mobile phase; flow velocity: 1.0 mL min^{-1}; detector: UV at 364 nm; sample size: 20 μl. In this research, experiments were performed in triplicate and the results were presented as average values.

3. Computational details

The removal efficiency and equilibrium adsorption quantity were calculated

according to Eqs. (1) and (2), respectively.

$$\text{n (\%)} = \frac{(C_0 - C_e)}{C_0} \times 100 \qquad (1)$$

$$q_e = \frac{(C_0 - C_e)V}{W} \qquad (2)$$

where n is the removal efficiency of OTC from the solution; q_e (mg g^{-1}) is the adsorption capacity in the adsorbent at equilibrium point; C_e and C_0 (mg L^{-1}) are the equilibrium and initial OTC concentrations of solution respectively; V (L) is the volume of aqueous solution and W (g) is the dosage of adsorbent.

The adsorption kinetics for adsorbent has been previously described by the pseudo-first-order and pseudo-second-order reactions. The linearized integral forms of both models are shown as Eqs. (3) and (4) respectively:

$$\log(q_e - q_t) = \log q_e - \frac{k_1}{2.303} t \qquad (3)$$

$$\frac{t}{q_t} = \frac{1}{k_2 q_e^2} + \frac{t}{q_e} \qquad (4)$$

where k_1 (min^{-1}) and k_2 (mg^{-1} min^{-1}) are the pseudo-first-order and pseudo-second-order rate constants respectively; q_e and q_t (mg g^{-1}) are the amounts of OTC adsorbed at equilibrium and at time (t) respectively.

The sorption equilibrium data are commonly correlated with Langmuir (Eqs. (5) and (6)) and Freundlich (Eq. (7)) models, as have been shown in previous reports.

$$\frac{C_e}{q_e} = \frac{1}{q_{max} k} + \frac{1}{q_{max}} C_e \qquad (5)$$

$$R_L = \frac{1}{1 + kC_0} \qquad (6)$$

$$\log q_e = \log K_f + \frac{1}{n} \log C_e \qquad (7)$$

Where q_e (mg g^{-1}) is the adsorption capacity at equilibrium; C_0 and C_e (mg L^{-1}) are

the initial and equilibrium OTC concentrations; k (L mg^{-1}) is the Langmuir constant related to the adsorption energy; and q_{max} (mg g^{-1}) is the theoretical adsorption capacity. The basic characteristic can be described by R_L, a dimensionless equilibrium parameter of 0<RL<1 indicates favorable adsorption, R_L=1 indicates linear adsorption and R_L>1 indicates unfavorable adsorption. K_f (mg g^{-1}) (L mg^{-1})$^{1/n}$ is the Freundlich constant, while n expresses the aggregation number of OTC molecules.

4. Characterization of Fe-MCM-41-A

Supplement characterization of Fe-MCM-41-A including BET， XRD and

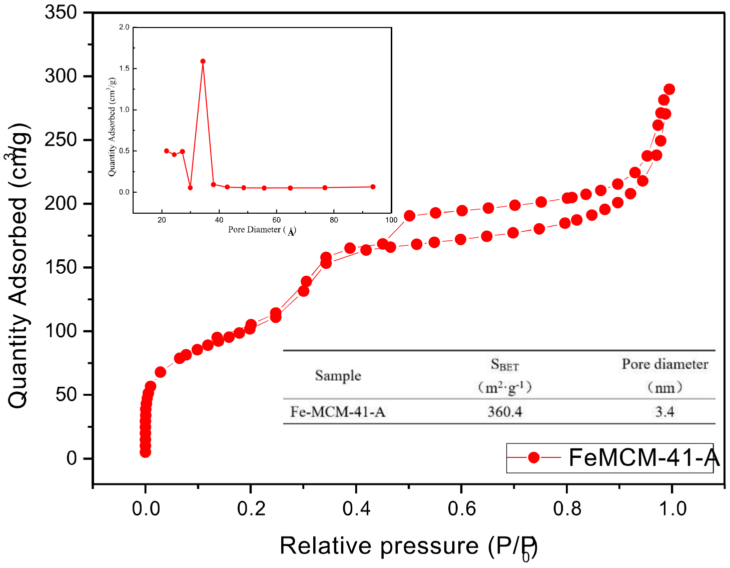

Fig. 1S N_2 adsorption/desorption isotherms and inset with PSD curve of Fe-MCM-41-A

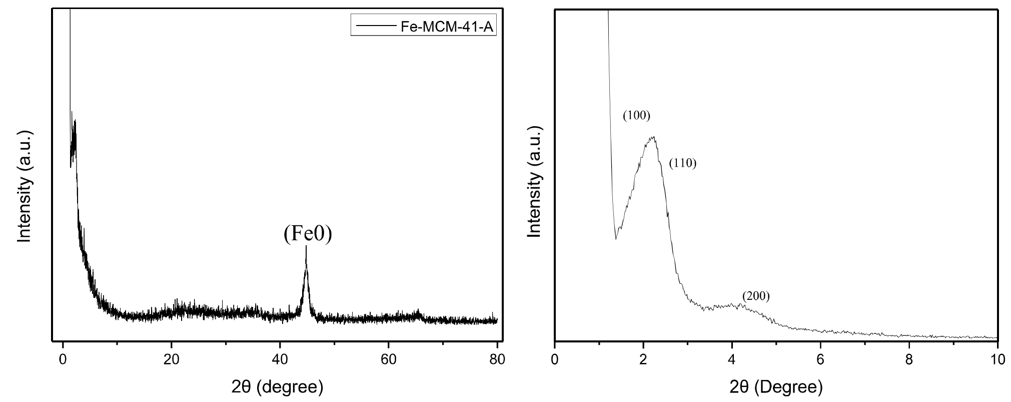

(a) (b)

Fig. 2S X-ray diffraction of Fe-MCM-41-A: (a) 0.7-80° and (b) 0.7-10°

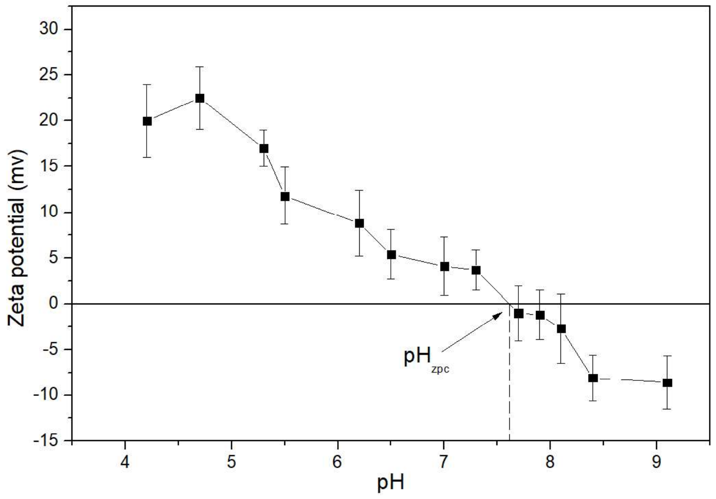

Fig.3S pH-Zeta of Fe-MCM-41-A

5. The physicochemical property of OTC

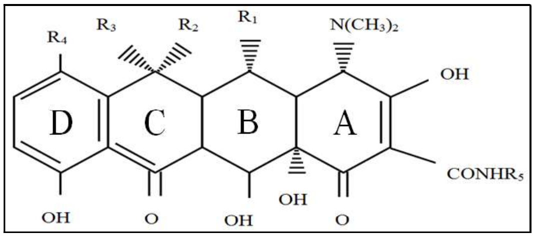

(a)

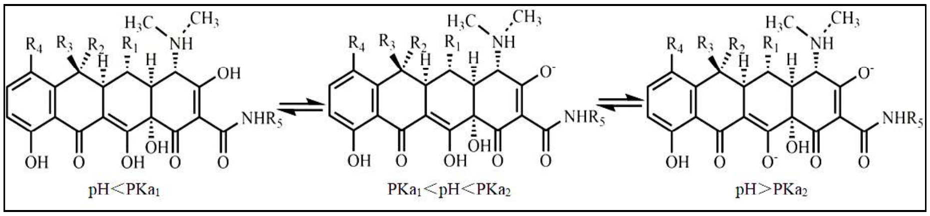

(b)

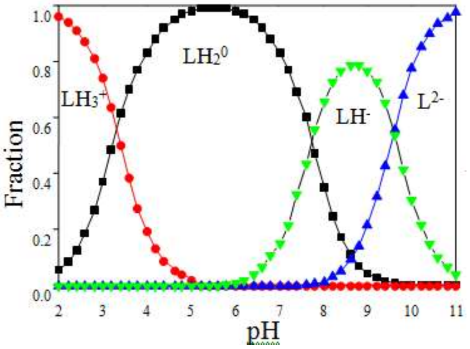

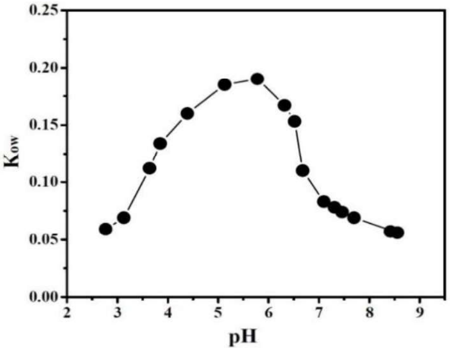

(c) (d)

Fig. 4S (a) The molecular formula for TCs, (b) Surface charge of TCs, (c) Molecular specie of TCs, (d) *Kow* of TCs with pH

6. Regeneration Experiment

The exhausted Fe-MCM-41-A was regenerated by ultrasonic vibration (22.5 kHz) in 100 mL of NaOH solution (0.01 M) for 30 min. After the samples were washed with water, $NaBH_4$ solution was added dropwise and the washing procedure was repeated as described in section 2.2. The final adsorbent was then dried in vacuum environment at 50°C for 10 h. The regenerated adsorbent was transferred into 200 mL of 100 mg L^{-1} OTC solution for re-adsorption under the optimum adsorption conditions. This adsorption-desorption cycle was repeated five times in order to study the reusability of Fe-MCM-41-A.
